# *Circ‐E‐Cad* encodes a protein that promotes the proliferation and migration of gastric cancer via the TGF‐β/Smad/C‐E‐Cad/PI3K/AKT pathway

**DOI:** 10.1002/mc.23491

**Published:** 2022-12-01

**Authors:** Fanying Li, Hongxing Tang, Shaoji Zhao, Xinya Gao, Lixuan Yang, Jianbo Xu

**Affiliations:** ^1^ Department of Neurosurgery The First Affiliated Hospital of Sun Yat‐sen University Guangzhou People's Republic of China; ^2^ Department of Gastrointestinal Surgery The First Affiliated Hospital of Sun Yat‐sen University Guangzhou People's Republic of China

**Keywords:** C‐E‐Cad, EMT, gastric cancer, migration, proliferation

## Abstract

Accumulating studies indicate that circular RNAs (circRNAs) play critical roles in cancer progression. Most of them have been reported to act as microRNA sponges or interact with RNA‐binding proteins; however, their full range of functions remains largely unclear. Recently, an increasing number of circRNAs have been found to encode proteins. C‐E‐Cad, a protein encoded by circular E‐cadherin (*circ‐E‐Cad*), has been shown to have a great influence in the progression of glioblastoma, but its specific role in gastric cancer (GC) is unclear. Here, we found that both *circ‐E‐Cad* and C‐E‐Cad were upregulated in GC cell lines and GC tissues compared with a human gastric epithelial cell line (GES‐1) and normal tissues. Knockdown of *circ‐E‐Cad* suppressed GC cell line proliferation and metastasis in vitro and in vivo, whereas overexpression of C‐E‐Cad had the opposite effects. Immunoblotting revealed that C‐E‐Cad exerted tumor‐promoting functions by regulating the PI3K/AKT pathway. A rescue experiment showed that C‐E‐Cad but not *circ‐E‐Cad* was the executor of protumor biological functions. In addition, we demonstrated that the C‐E‐Cad expression level could have been increased by the TGF‐β/Smad pathway. In summary, our results indicated that the TGF‐β/Smad pathway could increase the expression of C‐E‐Cad to regulate GC cell proliferation, migration, and epithelial‐mesenchymal transition by affecting PI3K/AKT signaling.

## 1 INTRODUCTION

Gastric cancer (GC) is one of the most common cancers in the world and is the third most common cause of death.^1^ Patients with an early diagnosis of GC could have a good curative effect after surgical treatment, but most GC patients have no chance of surgery, as they are diagnosed in the middle and advanced stages. Therefore, the development of novel treatment regimens is urgently needed to improve the survival of GC patients. At present, targeted therapy has become the main treatment for various malignant tumors, and molecular targeted drug therapy for GC patients has made some progress.^2^ However, satisfactory treatment results have not yet been achieved.

Circular RNAs (circRNAs) are covalently closed RNA transcripts and have long been considered noncoding RNAs; they are generally expressed at lower levels than their associated linear mRNAs. They have been shown to act as microRNA sponges or interact with RNA‐binding proteins (RBPs) in a variety of diseases.^3^, ^4^, ^5^ Recently, an increasing number of circRNAs have been found to encode proteins or peptides.^6^, ^7^, ^8^, ^9^ At present, few studies have investigated proteins or peptides from circRNAs in GC, and the underlying mechanism by which circRNAs or their translation products modulate GC has not yet been fully elucidated.

In our study, we found that circular E‐cadherin (*circ‐E‐Cad*) was abnormally expressed between GC tissues and adjacent normal tissues. We also showed that the novel protein, C‐E‐Cad, reported in our earlier studies^6^ was upregulated in GC tissues and GC cell lines. C‐E‐Cad was able to promote the proliferation and migration of GC both in vitro and in vivo. Furthermore, we demonstrated that the C‐E‐Cad protein level could have been increased by the TGF‐β/Smad pathway to upregulate the PI3K/AKT pathway. The TGF‐β/Smad/C‐E‐Cad/PI3K/AKT regulatory axis may offer a novel therapeutic target for patients with GC.

## 2 MATERIALS AND METHODS

### 2.1 Sample collection

GC tissue samples and paired adjacent normal tissue samples were collected from 100 cases treated in First Affiliated Hospital of Sun Yat‐sen University from 2017 to 2021. All patients had not received any radiotherapy or chemotherapy before surgery. GC tumor tissue and paired adjacent normal tissue obtained from patients were identified by experienced pathologists. Tumor tissue samples were immediately preserved in RNA solution after surgical resection. This study was approved by the Institutional Review Board (IRB) of the First Affiliated Hospital of Sun Yat‐sen University and agreed to by all of the patients.

### 2.2 Cell lines and cell culture

The GC cell lines (AGS, SGC‐7901, KATO, MGC803, and BGC823), and a human gastric epithelial cell line (GES‐1) were bought from the Type Culture Collection of the Chinese Academy of Sciences. All cell lines were grown in RPMI 1640 (GIBCO) supplemented with 10% certified fetal bovine serum (FBS; HyClone; GE Healthcare Life Sciences), 100 U/ml streptomycin (Gibco; Thermo Fisher Scientific), and 100 U/ml penicillin (Gibco; Thermo Fisher Scientific) and maintained in a thermostatic incubator with 5% CO_2_ at 37°C before use.

### 2.3 Establishment of stable cell lines and transfection

For the establishment of stable overexpressing and stable knockdown of GC cell lines, Lentiviral vectors expressing *circ‐E‐Cad*, C‐E‐Cad ORF, sh‐*circ‐E‐Cad, circ‐E‐Cad*‐ATG‐mut, or sh‐TGF‐beta RII were cotransfected with the packaging vectors psPAX2 (Addgene) and pMD2G (Addgene) into HEK293T cells for lentivirus production using Lipofectamine 3000 (Thermo Fisher Scientific) according to the manufacturer's instructions. To establish stable cell lines, GC cells were transduced by using the above lentiviruses with polybrene (8 mg ml^−1^; Sigma). After incubating for 72 h, cells were selected with 2 mg ml^−1^ puromycin for 3 days. Detail information of regarding plasmids was showed at follow:

sh‐circ‐E‐Cad: shRNA1: AACAGAAAATAACGAACCTCT; sh‐circ‐E‐Cad: shRNA2: ATAACGAACCTCTGTGATGGA;

sh‐TGF‐beta RII: shRNA: CTGCAAGATACATGGCTCC; Smad2/3‐Mut: Smad2/3 Thr8Ala.

### 2.4 RNA fluorescence in situ hybridization (FISH)

Cells were incubated at 37°C in a solution containing 50% formamide, 2× SSC, 0.25 mg ml^−1^ *Escherichia coli* transfer RNA, 0.25 mg ml^−1^ salmon sperm DNA (Life Technologies), 2.5 mg ml^−1^ BSA (Roche), and 125 nM fluorescently labeled junction probe (Generay). After 12 h, the cells were washed and mounted in ProLong Gold (Life Technologies) and incubated overnight at room temperature. Confocal microscopy imaging (Olympus FV100) was then performed.

### 2.5 Cell counting kit‐8 (CCK‐8) assay, colony formation assay (CFA), and EDU assay

GC cells proliferation was tested by using the CCK‐8 assay, CFA, and EDU assay according to the manufacturer's instructions. The CCK‐8 reagent (DOJINDO) was administered to 96‐well plates seeded at a density of 1 × 10^3^ cells per well, and absorbance was measured at 450 nm using a microplate reader (BioTek). CFA, GC cells were seeded into six‐well plates at 1 × 10^3^ per well and cultured in RPMI 1640 containing 10% FBS for 14 days. Then, the cells were fixed with methanol and stained with 0.5% crystal violet for 10 min. EdU assay Kit (RiboBio), 2 × 10^4^ GC cells per well were seeded in 96‐well plates. After 24 h, staining the GC cells by following the manufacturer's instructions. The proportion of cells incorporating EdU was detected and imaged by a fluorescence microscope (Leica).

### 2.6 Transwell migration assays

Migration was determined by Transwell plates (Corning). A total of 5 × 10^4^ cells were resuspended in 250 µl of serum‐free medium and added to the upper chamber, while the lower chamber was filled with 500 µl of complete medium. The chambers were then incubated for 48 h (5% CO_2_, 37°C). Cells in the upper chambers were fixed with methanol and stained with 0.1% crystal violet. Migrated cells were counted in three randomly selected microscopic fields.

### 2.7 RNA isolation and quantitative real‐time polymerase chain reaction (qRT‐PCR)

Total RNA from GC tissues and cell lines was extracted using TRIzol (Invitrogen). qRT‐PCR was used to detect the expression levels of *circ‐E‐Cad*. Glyceraldehyde 3‐phosphate dehydrogenase (GAPDH) was used for normalization. The primer sequences were as follows: *circ‐E‐Cad*: Forward, 5′‐GTG GGC CAG GAA ATC ACA TC‐3′ and Reverse, 5′‐TCA CAT CAT CGT CCG CGT CT‐3′; and GAPDH: Forward, 5′‐CCA GGT GGT CTC CTC TGA CTT‐3′, and Reverse, 5′‐GTT GCT GTA GCC AAA TTC GTT GT‐3′.

### 2.8 Western blot assay

Radioimmunoprecipitation buffer (Beyotime) was used to extract total protein from GC tissues and cell lines. Protein concentrations were quantified with a BCA Protein Assay Kit (Thermo Fisher Scientific). The primary antibodies used in this study included an antibody against C‐E‐Cad (1:1000; This study), E‐Cad (1:1000; Abcam), N‐Cad (1:1000; AbcamK), Snail (1:1000; Abcam), Slug (1:1000; Abcam), Vimentin (1:1000; Abcam), p‐AKT^S473^ (1:1000; Abcam), p‐AKT^T308^ (1:1000; Abcam), p‐PDK1 (1:1000; Abcam), AKT (1:1000; Abcam), PDK1 (1:1000; Abcam), p‐Smad2/3 (1:1000; Abcam), Smad2/3 (1:1000; Abcam). β‐actin (1:5000; Sigma‐Aldrich) was used as a control.

### 2.9 Immunohistochemistry (IHC)

Formalin and paraffin were used to fix and embed the isolated GC tissues, respectively. Then, tissues were cut into 5 mm thick. Immunochemical staining was performed following the standard protocol. Anti‐C‐E‐Cad (1:100; This study).

### 2.10 Xenograft and peritoneal dissemination model

Four weeks old female BALB/c nude mice were randomly assigned to experimental groups for all the experiments. For the xenograft model, *circ‐E‐Cad* knockdown, C‐E‐Cad ORF overexpression, and control GC cells (5 × 10^6^) resuspended in 100 µl of PBS were injected in the flank of nude mice (5 mice/group). These mice were killed 25 days after tumor cell implantation. At autopsy the tumor was removed. For peritoneal dissemination model, *circ‐E‐Cad* knockdown and control GC cells (2 × 10^6^) in 100 µl PBS were injected into the peritoneal cavity. Peritoneum metastasis was examined and recorded when mice were killed at 14 days after injection.

### 2.11 Statistical analysis

All data were analyzed by SPSS 22.0 software (SPSS) and GraphPad Prism 8.0 (GraphPad). Measurement data are presented by means ± standard deviation. Subsequently, a *t*‐test was applied for comparison between two groups, while a one‐way analysis of variance was performed for comparisons among more than two groups. The difference with *p < 0.05, **p < 0.01, ***p < 0.001, or ****p < 0.0001 was considered statistically significant.

## 3 RESULTS

### 3.1 *Circ‐E‐Cad* overexpression in GC cells and tissues

**Figure 1:**
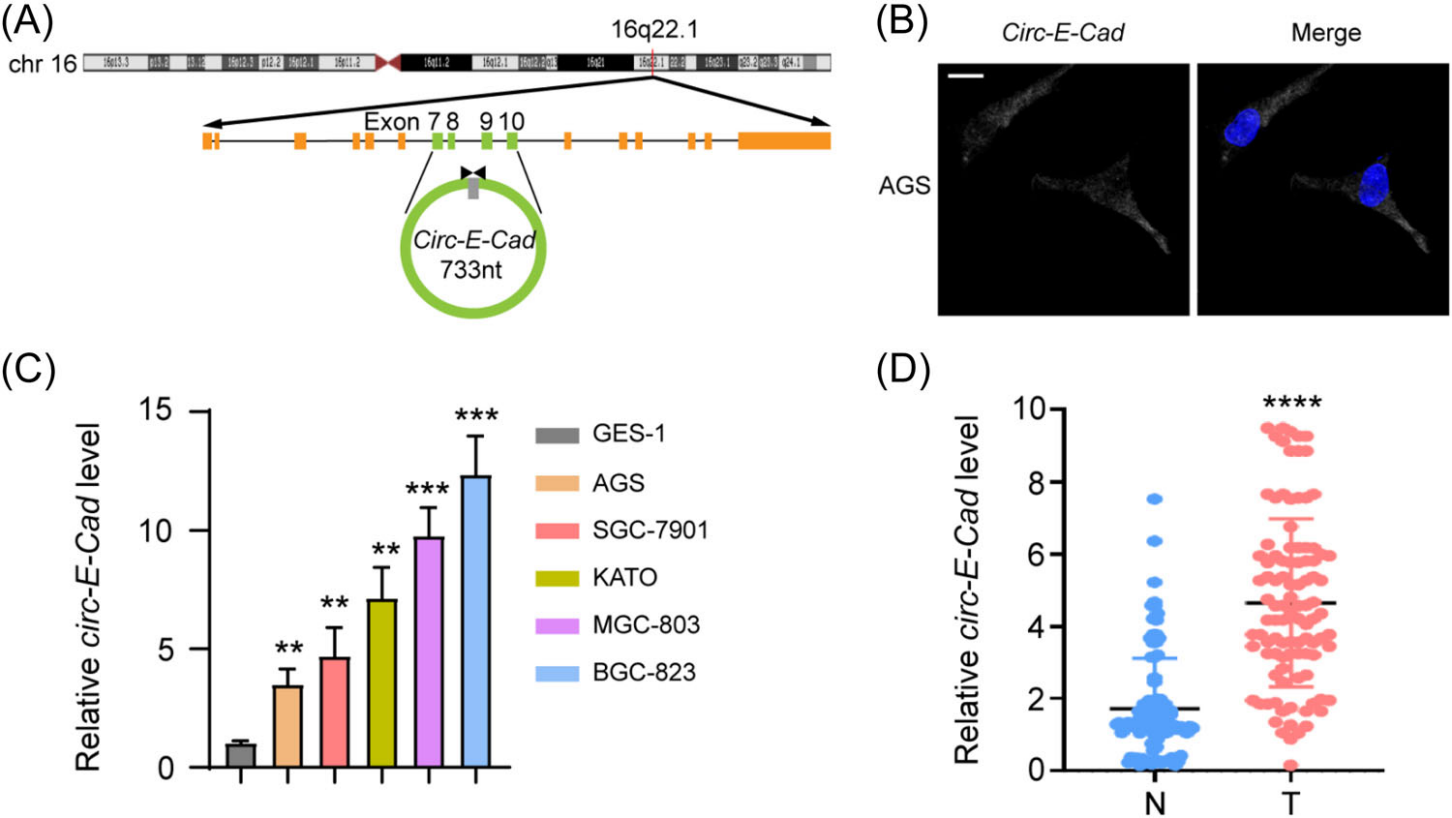
*Circ‐E‐Cad* is overexpression in GC cells and tissues. (A) The head‐to‐tail splicing of *circ‐E‐Cad*. (B) Fluorescence in situ hybridization with junction‐specific probe was used to decide the localization of *circ‐E‐Cad*. Scale bar, 20 µm. (C, D) Relative *circ‐C‐E‐Cad* RNA level of GES‐1 and GC cell lines, or of GC clinical samples and paired adjacent normal tissues in a cohort of 100 GC patients. Error bars represent three independent experiments (**p < 0.01; ***p < 0.001; ****p < 0.0001). GC, gastric cancer; GES‐1, gastric epithelial cell line. [Color figure can be viewed at wileyonlinelibrary.com]

It has been reported that 30%−40% of hereditary diffuse‐type gastric cancer patients carry *CDH1* mutations.^1^ *CDH1* encodes E‐cadherin, which has proven to inhibit the progression of GC.^10^ *Circ‐E‐Cad*, derived from exons 7−10 of its parental gene *CDH1* (Figure 1A), has the opposite expression pattern as E‐Cadherin mRNA in glioblastoma (GBM).^6^ To further validate the characteristics of *circ‐E‐Cad* in GC cells, we performed FISH and qRT‐PCR to determine the distribution of *circ‐E‐Cad* in GC cells. We found that circ‐E‐Cad was localized in the cytoplasm (Figure 1B), and the relative expression of *circ‐E‐Cad* was significantly higher in GC cell lines than in a human GES‐1 (Figure 1C). We also found *circ‐E‐Cad* to be significantly highly expressed in 100 paired GC tissues (Figure 1D). These findings indicated that *circ‐E‐Cad* was upregulated in GC cell lines and tissues, which suggests that it might be a useful tumor marker in GC.

### 3.2 C‐E‐Cad overexpression in GC cells and tissues

**Figure 2:**
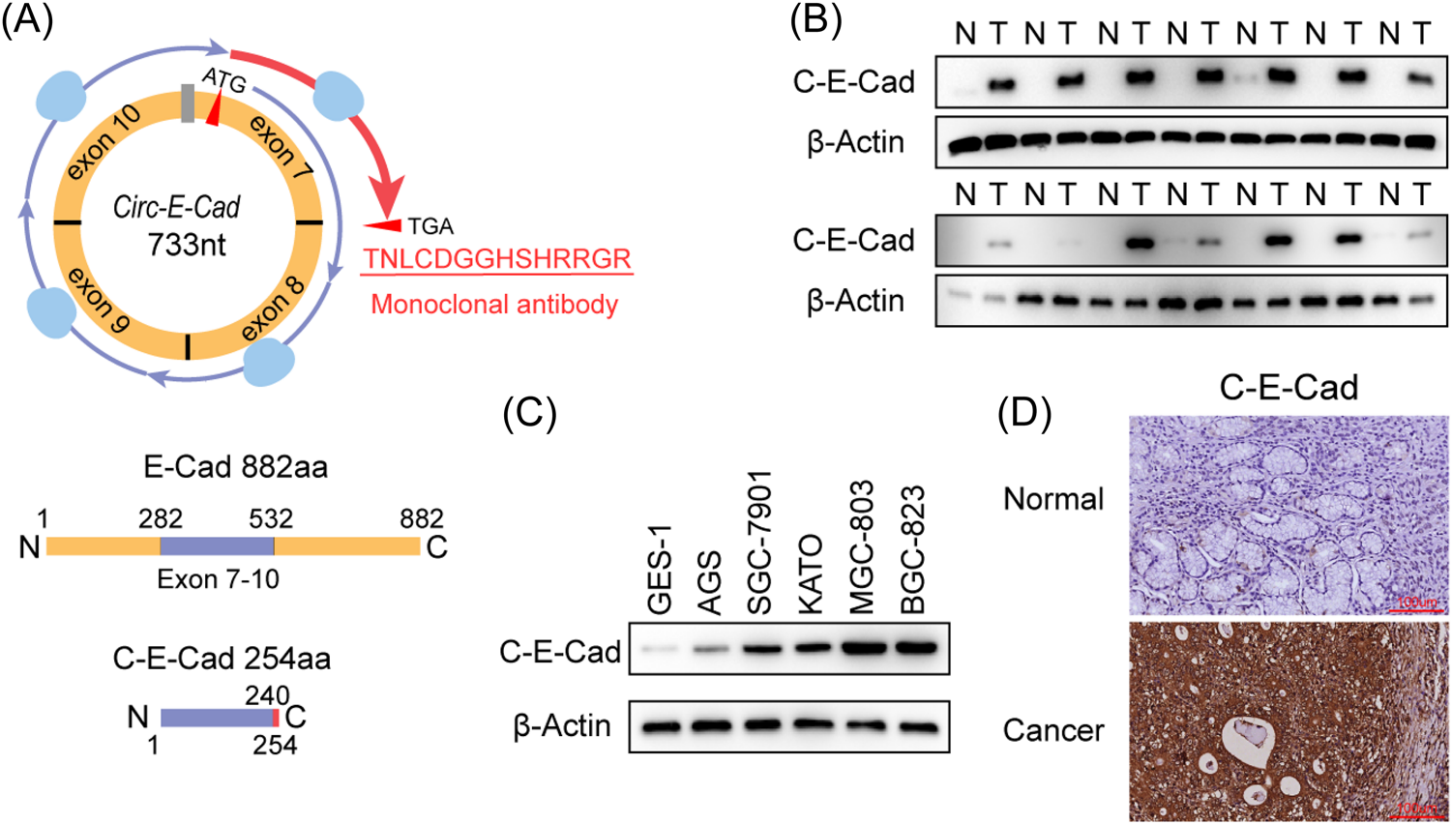
C‐E‐Cad is overexpression in GC cells and tissues. (A) Illustration of the C‐E‐Cad protein encoded by *circ‐E‐Cad* RNA. The C terminus of C‐E‐Cad is translated by the second‐round read (showing in red). A monoclonal mouse antibody was generated against the indicated C‐terminal sequences. (B) IB of C‐E‐Cad expression in 14 randomly selected GC tissues (T) and their paired normal tissues (N). (C) IB of C‐E‐Cad expression in GES‐1 and GC cell lines. (D) IHC images of C‐E‐Cad expression in GC and normal tissues. Scale bar, 100 µm. GC, gastric cancer; GES‐1, gastric epithelial cell line; IB, immunoblotting; IHC, immunohistochemistry. [Color figure can be viewed at wileyonlinelibrary.com]

It has been shown that *circ‐E‐Cad* can encode a 254‐amino acid protein, C‐E‐Cad (Figure 2A).^6^ Thus, we used the self‐preparing antibody to detect the C‐E‐Cad expression level by immunoblotting (IB) in paired cancerous/adjacent normal tissue >2 cm away in 14 randomly selected GC patients and in 5 GC cell lines compared with GES‐1. Consistently, C‐E‐Cad protein expression was also significantly higher in both GC tissues and GC cell lines (Figure 2B,C). This was further confirmed by IHC in paired cancerous/adjacent normal tissue from 100 GC patients (Figure 2D). These results suggested that C‐E‐Cad is an oncogenic protein and may serve as a novel tumor marker for GC.

### 3.3 C‐E‐Cad promotes GC cell proliferation, migration, and epithelial‐mesenchymal transition (EMT)

**Figure 3:**
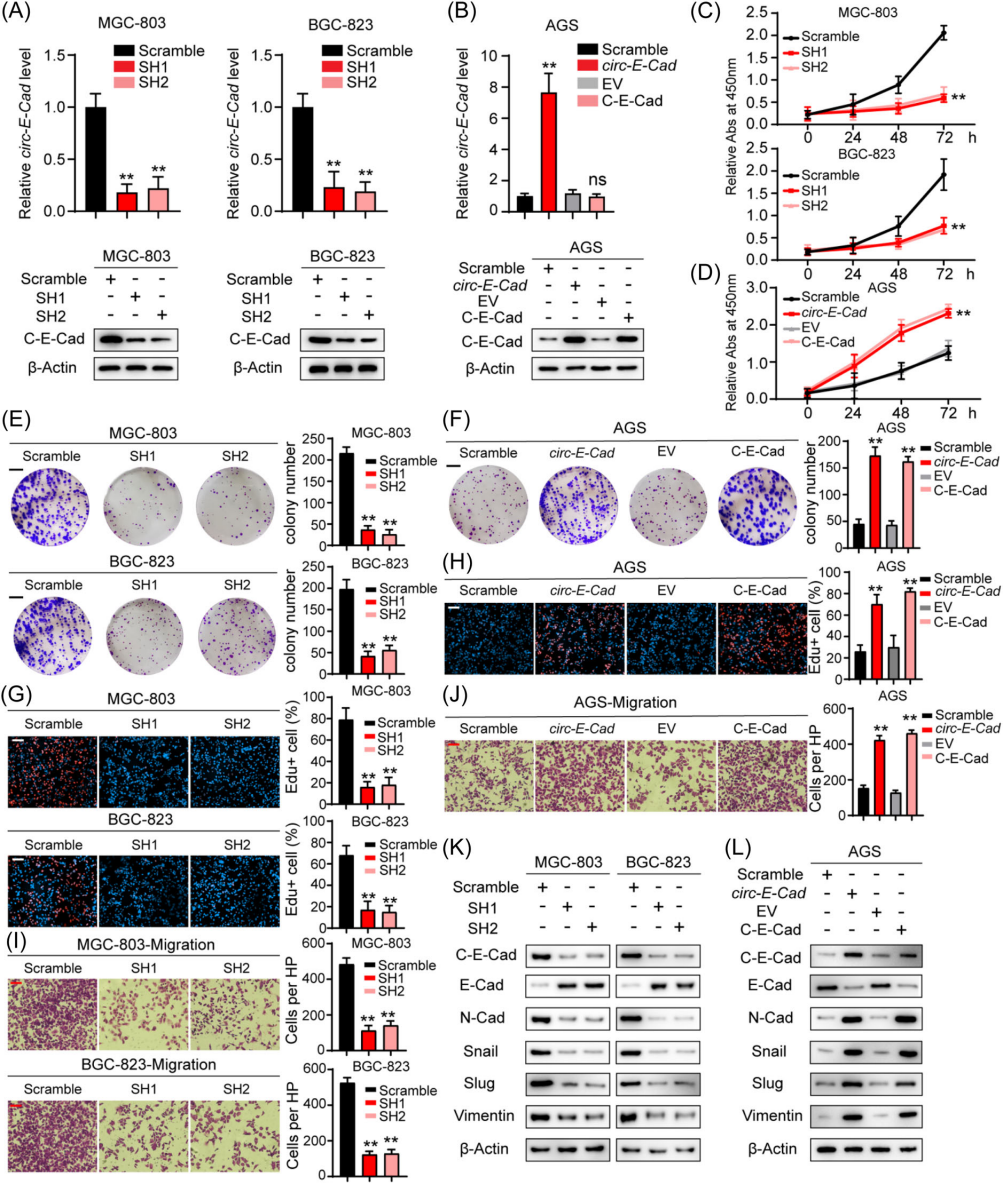
C‐E‐Cad promotes GC cells proliferation, migration and EMT. (A, B) qRT‐PCR analysis of *circ‐E‐Cad* RNA level and IB analysis of C‐E‐Cad protein level in MGC‐803, BGC‐823, and AGS cell lines transfected with Scramble, sh‐*circ‐E‐Cad*, *circ‐E‐Cad*, and C‐E‐Cad ORF, respectively (**p < 0.01). (C−H) CCK8, colony formation, and EDU assays in MGC‐803, BGC‐823, and AGS cell lines transfected with Scramble, sh‐*circ‐E‐Cad*, *circ‐E‐Cad*, and C‐E‐Cad ORF, respectively. Scale bar, 100 µm (**p < 0.01). (I, J) Transwell migration assays in MGC‐803, BGC‐823, and AGS cell lines transfected with Scramble, sh‐*circ‐E‐Cad*, *circ‐E‐Cad*, and C‐E‐Cad ORF, respectively. Scale bar, 100 µm (**p < 0.01). (K, L) IB of EMT marker proteins expression level in MGC‐803, BGC‐823, and AGS cell lines transfected with Scramble, sh‐*circ‐E‐Cad*, *circ‐E‐Cad*, and C‐E‐Cad ORF, respectively. CCK8, cell counting kit‐8; EMT, epithelial‐mesenchymal transition; GC, gastric cancer; IB, immunoblotting; qRT‐PCR, quantitative real‐time polymerase chain reaction. [Color figure can be viewed at wileyonlinelibrary.com]

In GC cell lines, C‐E‐Cad was highly expressed in MGC‐803 and BGC‐823 cells and expressed at low levels in AGS cells (Figure 2C). Therefore, we transfected MGC‐803 and BGC‐823 cells with shRNA‐targeting *circ‐E‐Cads* (MGC‐803 SH1, MGC‐803 SH2, BGC‐823 SH1, and BGC‐823 SH2) and transfected AGS cells with functional *circ‐E‐Cad* (AGS circ‐E‐Cad) and C‐E‐Cad ORF (AGS C‐E‐Cad). qRT‐PCR and IB confirmed that the expression of both *circ‐E‐Cad* and C‐E‐Cad was effectively modulated in MGC‐803, BGC‐823, and AGS cells (Figure 3A,B). Functionally, the CCK‐8 assay, CFA, and EdU assay showed that knockdown of *circ‐E‐Cad* significantly decreased cell proliferation in MGC‐803 and BGC‐823 cells, whereas overexpression of both *circ‐E‐Cad* and C‐E‐Cad ORF significantly increased cell proliferation in AGS cells (Figure 3C−H). As expected, using Transwell migration assays, we also obtained similar results in MGC‐803, BGC‐823, and AGS cells (Figure 3I,J). We further explored the function of C‐E‐Cad in EMT in GC. IB indicated that downregulation of C‐E‐Cad inhibited EMT by reducing the mesenchymal markers N‐Cad, Snail, Slug and vimentin and upregulating the epithelial marker E‐cadherin, and overexpression of C‐E‐Cad significantly promoted EMT by the opposite change in those markers (Figure 3K,L). By overexpressing C‐E‐Cad‐ORF in MGC‐803 SH1/2 and BGC‐823 SH1/2 cells and overexpressing *circ‐E‐Cad* with C‐E‐Cad ORF ATG mutated in AGS cells, the rescue experiments suggested that C‐E‐Cad but not *circ‐E‐Cad* is the executor of protumor biological functions (Supporting Information: Figure S1A−F). In summary, these results demonstrated that C‐E‐Cad could significantly promote GC cell proliferation, migration, and EMT.

### 3.4 C‐E‐Cad upregulates the PI3K/AKT pathway in GC cells

**Figure 4:**
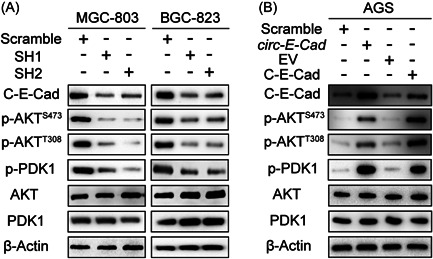
C‐E‐Cad upregulate PI3K/AKT pathway in GC cells. (A, B) IB of PI3K/AKT pathway proteins level in MGC‐803, BGC‐823, and AGS cell lines transfected with Scramble, sh‐*circ‐E‐Cad*, *circ‐E‐Cad*, and C‐E‐Cad ORF, respectively. GC, gastric cancer; IB, immunoblotting.

Next, we tried to elucidate the molecular mechanisms underlying the protumor function of C‐E‐Cad in GC cells. A preliminary study showed that C‐E‐Cad could promote the progression of GBM via the PI3K/AKT pathway.^6^ A large number of studies have shown that the PI3K/AKT pathway plays an important role in the progression of GC.^11^, ^12^, ^13^, ^14^ The PI3K/AKT pathway was detected by IB. Downregulated C‐E‐Cad in MGC‐803 and BGC‐823 cells decreased the expression of both p‐AKT and p‐PDK1, and upregulated C‐E‐Cad in AGS cells increased p‐AKT and p‐PDK1 expression (Figure 4A,B). By overexpressing C‐E‐Cad‐ORF in MGC‐803 SH1/2 and BGC‐823 SH1/2 cells and overexpressing *circ‐E‐Cad* with C‐E‐Cad ORF ATG mutated in AGS cells, the rescue experiments also suggested that C‐E‐Cad but not *circ‐E‐Cad* was effective (Supporting Information: Figure S2A,B). Moreover, MGC‐803 SH1 cell was successfully infected with constitutively active Akt (myr‐Akt) (Supporting Information: Figure S2C). And cell proliferation has been rescued by myr‐Akt (Supporting Information: Figure S2D). Then, we used MK2206 (Akt inhibitor) to treat the AGS cell and AGS cell transfected with *circ‐E‐Cad*. Cell proliferation inhibition rate showed that cells with forced expression of *circ‐E‐Cad* were more sensitive to AKT inhibitor (Supporting Information: Figure S2E). Together, these results indicated that C‐E‐Cad upregulated the PI3K/AKT pathway in GC cells.

### 3.5 TGF‐β increases C‐E‐Cad expression levels in GC cells

**Figure 5:**
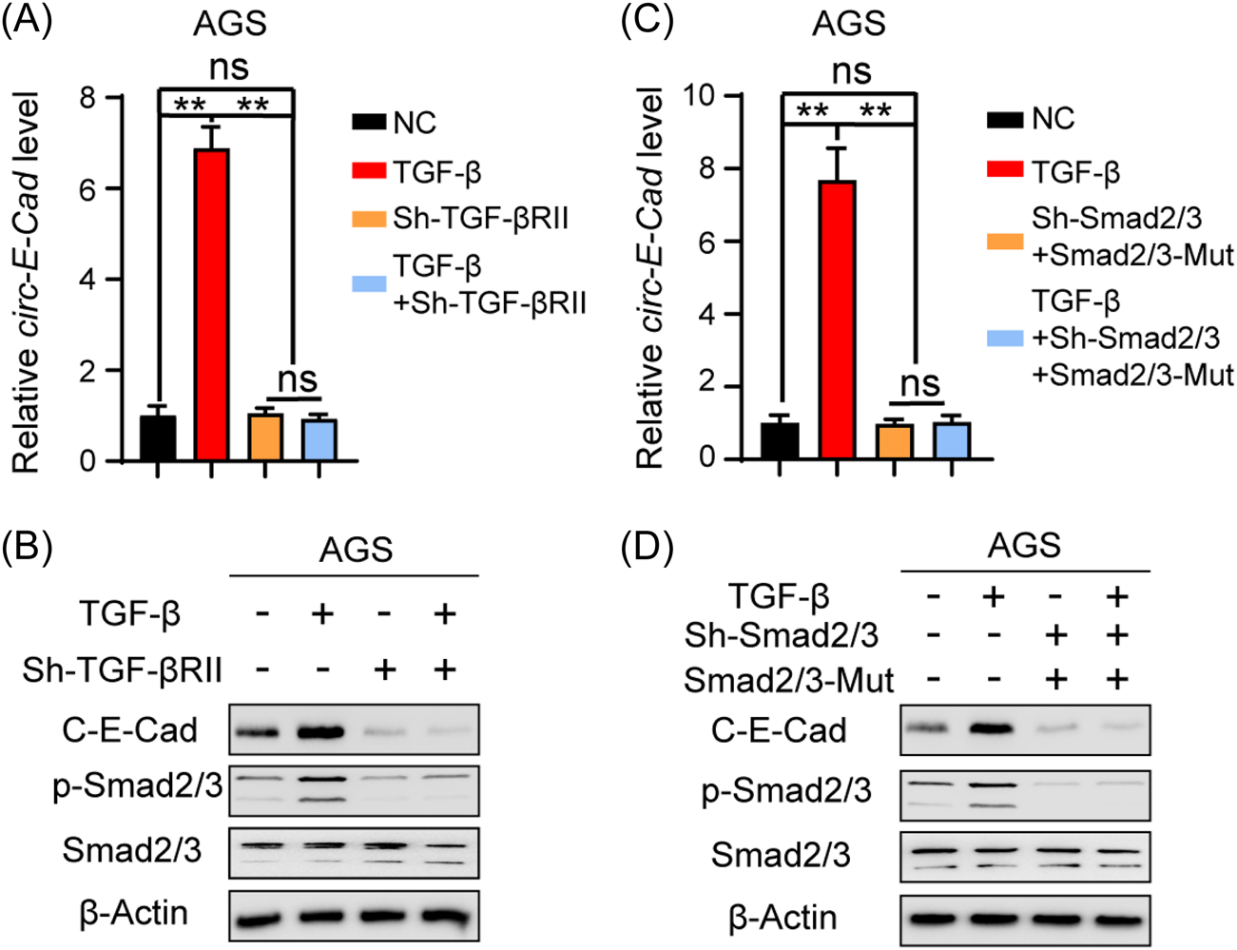
TGF‐β increases C‐E‐Cad expression level in GC cells. (A) qRT‐PCR of *circ‐E‐Cad* RNA expression level in AGS cells treated with TGF‐β, sh‐TGF‐βR, or NC, respectively or combine with each other (**p < 0.01). (B) IB of C‐E‐Cad, p‐Smad2/3, and Smad2/3 in AGS cells treated with TGF‐β, sh‐TGF‐βR, or NC, respectively or combine with each other. (C) qRT‐PCR of *circ‐E‐Cad* RNA expression level in AGS cells treated with TGF‐β, sh‐Smad2/3, Smad2/3 phosphorylation inactivation mutation (Smad2/3‐Mut), or NC, respectively or combine with each other (**p < 0.01). (D) IB of C‐E‐Cad, p‐Smad2/3, and Smad2/3 in AGS cells treated with TGF‐β, sh‐Smad2/3, Smad2/3 phosphorylation inactivation mutation (Smad2/3‐Mut), or NC, respectively or combine with each other. GC, gastric cancer; IB, immunoblotting; NC, negative control; qRT‐PCR, quantitative real‐time polymerase chain reaction. [Color figure can be viewed at wileyonlinelibrary.com]

The TGF‐β/Smad pathway plays a vital role in both the proliferation and EMT of GC cells.^15^ To explore the effect of TGF‐β on the expression of C‐E‐Cad, we used qRT‐PCR and IB to detect the expression levels of *circ‐E‐Cad* RNA and C‐E‐Cad protein in AGS cells and found that TGF‐β could increase both the *circ‐E‐Cad* RNA level and the C‐E‐Cad protein level (Figure 5A,B). Furthermore, to determine whether *circ‐E‐Cad* RNA was regulated by Smad2/3, we reexpressed a Smad2/3 phosphorylation inactivation mutation (Smad2/3‐Mut: Smad2/3 Thr8Ala) in sh‐Smad2/3 AGS cells and then treated them with TGF‐β. We found that both the expression levels of *circ‐E‐Cad* RNA and C‐E‐Cad protein no longer changed (Figure 5C,D). These results suggested that the TGF‐β/Smad pathway could increase the expression level of C‐E‐Cad in GC cells.

### 3.6 C‐E‐Cad regulates tumor growth and peritoneal dissemination in vivo

**Figure 6:**
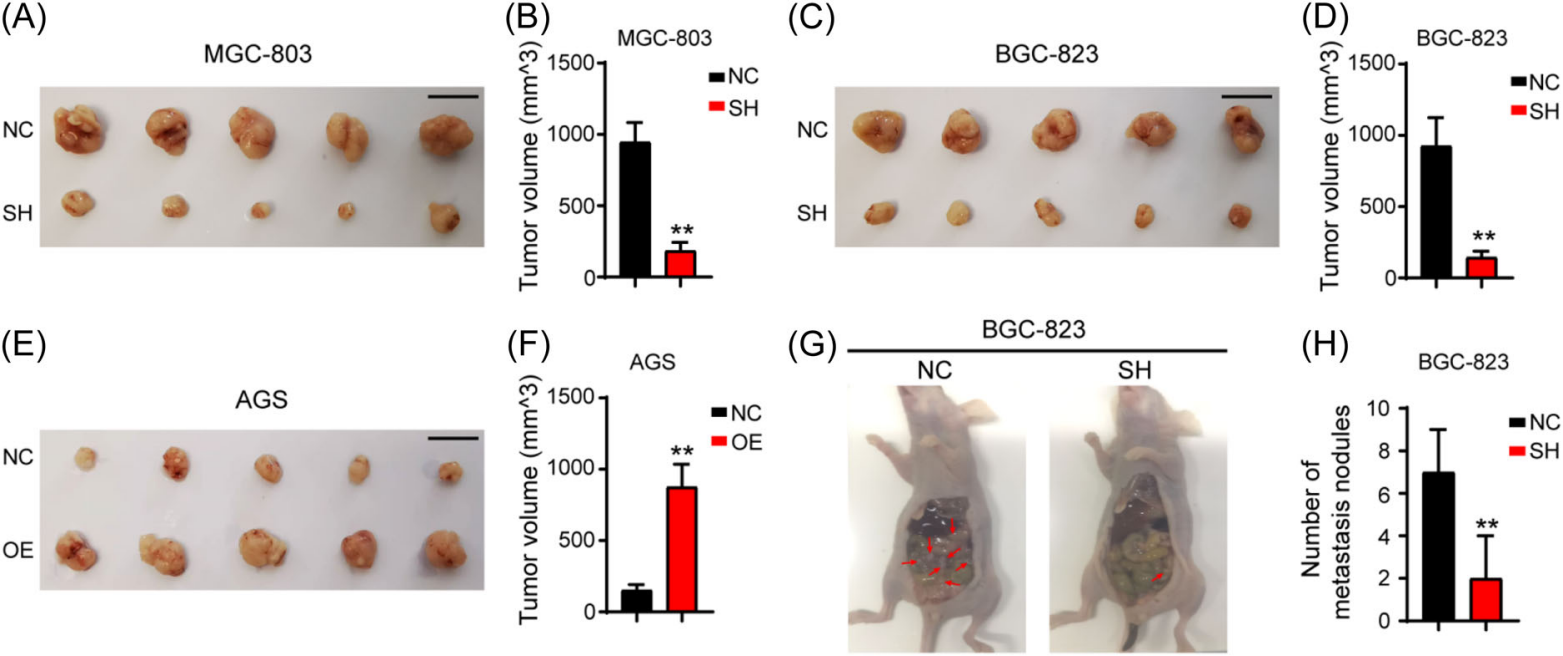
C‐E‐Cad regulate tumor growth and peritoneal dissemination in vivo. (A−D) The xenograft tumors induced by *circ‐E‐Cad* knockdown and empty vector in MGC803 and BGC823 cells were exhibited. Tumor volumes were analyzed. Scale bar, 1 cm (**p < 0.01). (E, F) The xenograft tumors induced by C‐E‐Cad overexpression and empty vector in AGS cells were exhibited. Tumor volumes were analyzed. Scale bar, 1 cm. (**p < 0.01). (G) Representative intestines of scattered tumors in nude mice. (H) The quantification of metastases in nude mice was counted (**p < 0.01). [Color figure can be viewed at wileyonlinelibrary.com]

The in vitro findings indicated that *circ‐E‐Cad* promoted GC growth and peritoneal dissemination in vivo. For the xenograft model, stable MGC‐803 and BGC‐823 cells with knockdown of *circ‐E‐Cad* and AGS cells with overexpression of C‐E‐Cad were injected into 4‐week‐old female nude mice. Tumor volume was measured using a Vernier caliper when mice were killed. We observed that the tumor volume in the *circ‐E‐Cad* knockdown group was markedly smaller than that in the control group (Figure 6A−D). The tumor volumes in the C‐E‐Cad overexpression group were markedly larger than those in the control group (Figure 6E,F). For the peritoneal dissemination model, 14 days after peritoneal cavity injection, the mice were killed. The results showed that knockdown of *circ‐E‐Cad* reduced the mesenteric metastatic nodules in the intestinal wall of nude mice (Figure 6G,H). These results showed that C‐E‐Cad could also regulate tumor growth and peritoneal dissemination in vivo.

## 4 DISCUSSION

Recently, mounting evidence has revealed that circRNAs are widely found in eukaryotes,^3^, ^16^ where they play an important role. In addition, many studies have demonstrated that circRNAs are potential biomarkers and therapeutic targets for multiple cancers.^6^, ^17^ Here, our data showed that *circ‐E‐Cad* RNA levels were higher not only in GC tissues than in normal gastric tissues but also in GC cells compared to GES‐1 cells. This finding suggests that *circ‐E‐Cad* might be a promising biomarker in GC. In addition, they act as miRNA sponges and interact with RBPs. They also have been found to encode proteins and peptides.^5^, ^6^, ^7^, ^8^, ^9^ Our previous research showed that *circ‐E‐Cad* encodes a 254‐amono acid protein, C‐E‐Cad.^6^ In the present study, we discovered that C‐E‐Cad protein levels were also higher in GC tissues and GC cells than in normal gastric tissues and GES‐1 cells. This also means that C‐E‐Cad is an oncogenic protein and may serve as a novel tumor marker for GC. However, this proposal is formulated based on a limited sample size. Further study is needed to confirm that C‐E‐Cad can be used as a prognostic biomarker.

The functions of these novel proteins in different cancers remain largely unknown. Here, CCK8, colony formation and EdU assays indicated that C‐E‐Cad promoted proliferation, migration and EMT of GC cells. Rescue experiments confirmed that C‐E‐Cad but not *circ‐E‐Cad* is the executor of protumor biological functions. Furthermore, qRT‐PCR and IB showed that C‐E‐Cad functions as an oncogenic protein by upregulating the PI3K/AKT pathway in GC cells. Given the central role of PI3K/AKT signaling in GC, targeting C‐E‐Cad might be a logical rationale for developing novel therapeutic strategies.

EMT is considered to be a key step in cancer metastasis.^18^, ^19^ EMT markers in GC are associated with poor patient outcomes.^20^ Moreover, emerging evidence shows that the TGF‐β/Smad pathway can induce EMT in GC.^15^ Here, the IB results showed that TGF‐β could directly increase *circ‐E‐Cad* RNA and C‐E‐Cad protein levels in GC cells. Rescue experiments confirmed that both the expression levels of *circ‐E‐Cad* RNA and C‐E‐Cad protein were regulated by the TGF‐β/Smad pathway in CG cells, which means that we could also block C‐E‐Cad by targeting the TGF‐β/Smad pathway.

In this study, *circ‐E‐Cad* shRNA showed a great therapeutic effect in xenografts and peritoneal dissemination models, with no severe side effects. We believe that in the future, C‐E‐Cad will be a new therapeutic target for GC in clinical trials.

## 5 CONCLUSIONS

Our study is the first to determine that C‐E‐Cad is highly expressed and promotes tumorigenesis and aggressiveness in GC. We found that the TGF‐β/Smad pathway could increase the expression level of C‐E‐Cad to regulate GC cell line proliferation, migration, and EMT by affecting PI3K/AKT signaling.

## AUTHOR CONTRIBUTIONS

Fanying Li, Xinya Gao, and Lixuan Yang designed this study and drafted the manuscript. Fanying Li and Hongxing Tang performed the cell experiment. Fanying Li and Shaoji Zhao performed the animal study. Shaoji Zhao and Jianbo Xu collected tissue samples and performed the statistical analysis. Jianbo Xu supervised the study and revised the manuscript. All authors read and gave final approval of the manuscript.

## CONFLICT OF INTEREST

The authors declare no conflict of interest.

## Supporting information

Supporting information.

Supporting information.

## Data Availability

The data used to support the findings of this study are available from the corresponding author upon request.
