# Supplementary material for: Circ‐E‐Cad encodes a protein that promotes the proliferation and migration of gastric cancer via the TGF‐β/Smad/C‐E‐Cad/PI3K/AKT pathway
Source: Mol Carcinog. 2022 Dec 1;62(3):360–8. doi: 10.1002/mc.23491 (PMC10107598; doi:10.1002/mc.23491)
Supplement: Supplementary file 2 — Supporting information. [file MC-62-360-s001.doc]

**Supplementary Figures**


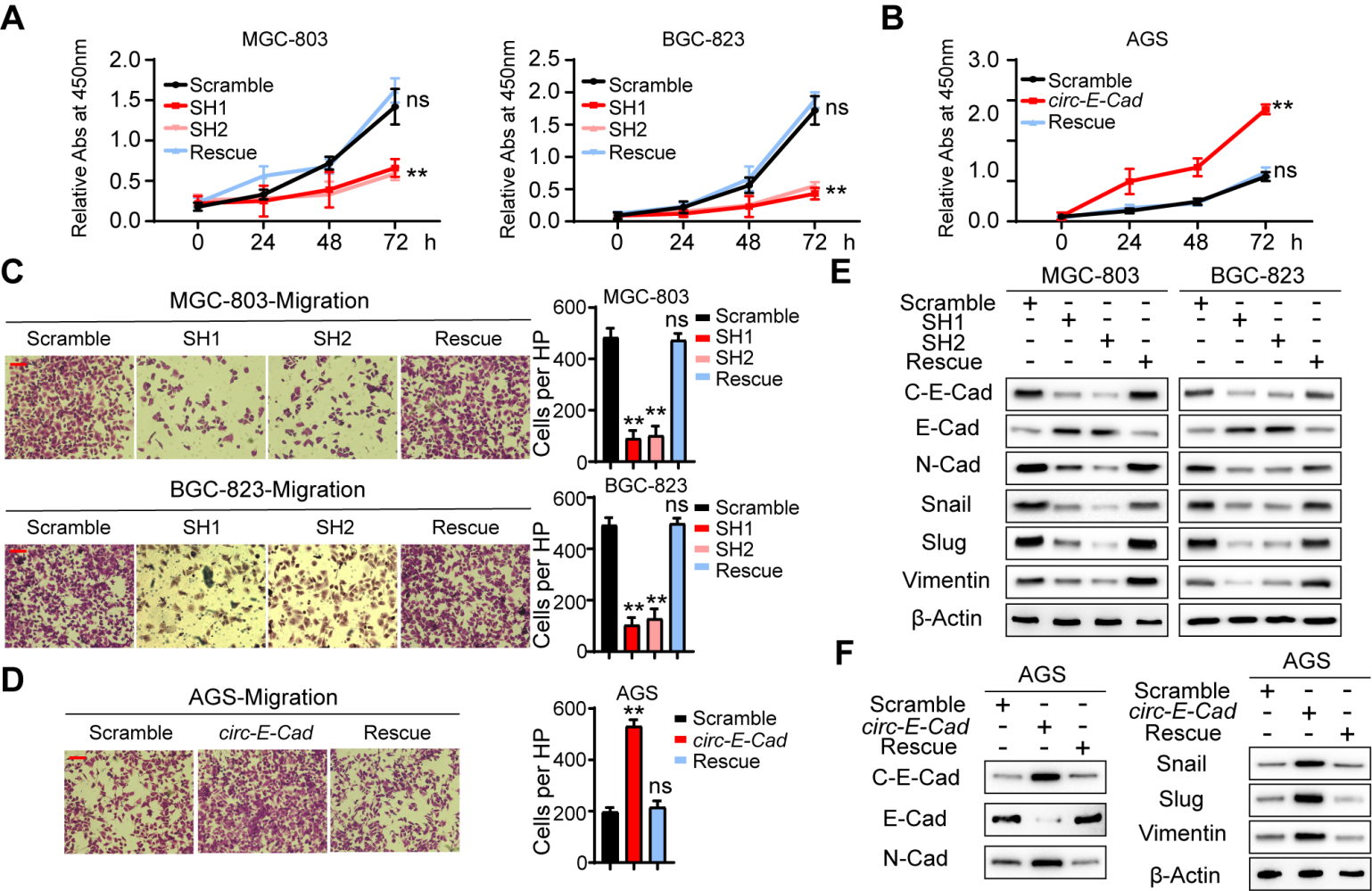


**Figure S1** C-E-Cad promotes GC cells proliferation, migration and EMT. A, CCK8 assay in MGC-803 and BGC-823 cell lines transfected with Scramble, sh-*circ-E-Cad*, and rescued by C-E-Cad ORF(**, *p<0.01*). B, CCK8 assay in AGS cell transfected with Scramble, *circ-E-Cad*, and rescued by *circ-E-Cad* ATG-mut (**, *p<0.01*). C, Transwell migration assays in MGC-803 and BGC-823 cell lines transfected with Scramble, sh-*circ-E-Cad*, and rescued by C-E-Cad ORF. Scale bar, 100 µm (**, *p<0.01*). D, Transwell migration assays in AGS cell transfected with Scramble, *circ-E-Cad*, and rescued by *circ-E-Cad* ATG-mut. Scale bar, 100 µm (**, *p<0.01*). E, IB of EMT marker proteins expression level in MGC-803 and BGC-823 cell lines transfected with Scramble, sh-*circ-E-Cad*, and rescued by C-E-Cad ORF. F, IB of EMT marker proteins expression level in AGS cell transfected with Scramble, *circ-E-Cad*, and rescued by *circ-E-Cad* ATG-mut.


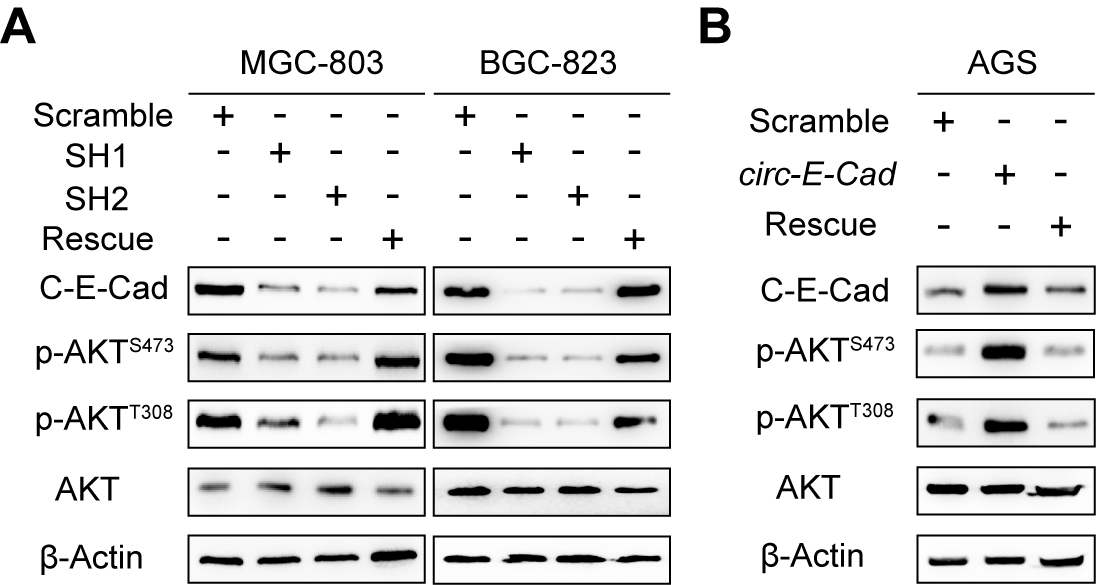


**Figure S2** C-E-Cad upregulate PI3K/AKT pathway in GC cells. A, IB of PI3K/AKT pathway proteins level in MGC-803 and BGC-823 cell lines transfected with Scramble, sh-*circ-E-Cad*, and rescued by C-E-Cad ORF. B, IB of PI3K/AKT pathway proteins level in AGS cell transfected with Scramble, *circ-E-Cad*, and rescued by *circ-E-Cad* ATG-mut.
